# Supplementary material for: Dolutegravir based antiretroviral therapy compared to other combined antiretroviral regimens for the treatment of HIV-infected naive patients: A systematic review and meta-analysis
Source: PLoS One. 2019 Sep 10;14(9):e0222229. doi: 10.1371/journal.pone.0222229 (PMC6736283; doi:10.1371/journal.pone.0222229)
Supplement: Attachment — Submitted filename: PONE-D-19-17880_Comments due 23 July 2019.docx [file pone.0222229.s009.docx]

PONE-D-19-17880	Accepted July 09 2019

Due July 23 2019

Submitted July 18 2019

Dolutegravir based antiretroviral therapy compared to other combined antiretroviral regimens for the treatment of HIV-infected naive patients: a systematic review and meta-analysis

Comments:

Define “naïve” in the Introduction

There are only two authors. Was there an adjudicator in the data extraction process in case of disagreements?

90 ***Assessment of risk of bias and Heterogeneity***

Below line 90

Put here gradings of heterogeneity as in I^2^ = 0% indicating no heterogeneity

Restrict mentioning RevMan more than twice

Did the authors perform tests for heterogeneity?

***Strategy for data synthesis***

104 We used aggregate data. The analysis has been conducted

Substitute the underlined with “was”

107 random effects analyses are undertaken and any differences

Substitute the underlined with “performed”

109 pre-specified subgroup analysis. We used risk difference as measure of effect. Review Manager 5.3 was used to analyze the data.

RevMan again?

Table 2 . legend

Include RD

203 adverse events and of adverse events requiring discontinuation of treatment was not significantly different in dolutegravir

What is the p-value for the “not significantly different “?

Kindly organize your Discussion so as to indicate summary of effects, clinical implications

Also mention of strengths and limitations of this study

156 Table 2 . Summary of the pooled outcome data. Results are provided for a 156 ll possible comparisons, and for

157 subgroups analyses

In column 5 of the P-values, the authors present several significant values

Did the authors correct for multiple comparisons?

The favours labels are opposite at the x-axis in Figures 3 and 5

Is this correct?

Suppl figures 1 and 2

Need to label DTG in legend

Supl Fig 4. Forest plot of comparison: DTG vs EFV, outcome: VL<50 48 wks

SINGLE 48 contributed 57.7% to the pooled RD, mention this in the text because it is an imbalance in weight contribution

Supl Fig 5. Forest plot of comparison: Dolutegravir vs comparators, outcome: VL<50 96 wks

The authors used random-effects here, why not fixed?

Supl Fig 6. Forest plot of comparison: DTG vs other according to baseline CD4 (cut off 200 CD4/ml) ,

outcome: % <50.

The authors used random-effects here even if the 9.3.1 plot is I^2^ = 0%. Please justify

Supl Fig 7. Forest plot of comparison: Dolutegravir vs comparators, outcome: Most common adverse

effects. Is the label favouring Dolutegravir same is DTG?
