# Supplementary material for: Dolutegravir based antiretroviral therapy compared to other combined antiretroviral regimens for the treatment of HIV-infected naive patients: A systematic review and meta-analysis
Source: PLoS One. 2019 Sep 10;14(9):e0222229. doi: 10.1371/journal.pone.0222229 (PMC6736283; doi:10.1371/journal.pone.0222229)
Supplement: Attachment — Submitted filename: response to reviewers.docx [file pone.0222229.s010.docx]

Journal Requirements:

1. When submitting your revision, we need you to address these additional requirements.

Please ensure that your manuscript meets PLOS ONE's style requirements, including those for file naming. The PLOS ONE style templates can be found at
<http://www.journals.plos.org/plosone/s/file?id=wjVg/PLOSOne_formatting_sample_main_body.pdf> and <http://www.journals.plos.org/plosone/s/file?id=ba62/PLOSOne_formatting_sample_title_authors_affiliations.pdf>

AU: OK

2. Please include captions for your Supporting Information files at the end of your manuscript, and update any in-text citations to match accordingly. Please see our Supporting Information guidelines for more information: <http://journals.plos.org/plosone/s/supporting-information>.

AU: OK

3. We note that you have included the phrase “data not shown” in your manuscript. Unfortunately, this does not meet our data sharing requirements. PLOS does not permit references to inaccessible data. We require that authors provide all relevant data within the paper, Supporting Information files, or in an acceptable, public repository. Please add a citation to support this phrase or upload the data that corresponds with these findings to a stable repository (such as Figshare or Dryad) and provide and URLs, DOIs, or accession numbers that may be used to access these data. Or, if the data are not a core part of the research being presented in your study, we ask that you remove the phrase that refers to these data.

AU: as suggested, we have removed the sentence, and specified that funnel plots were not performed because the low number of studies (lines 199-200)

4. Please confirm that you have included all items recommended in the PRISMA checklist including an assessment of publication bias using graphical methods (e.g. Funnel plot) and statistical methods (e.g. Egger’s test) as appropriate; details of reasons for study exclusions in the PRISMA flowchart and number of studies excluded for each reason; and the full electronic search strategy used to identify studies with all search terms and limits for at least one database.

AU: done

5. We noticed you have some minor occurrence of overlapping text with the following previous publication(s), which needs to be addressed:

<https://aidsinfo.nih.gov/contentfiles/lvguidelines/AdultandAdolescentGL.pdf>
<https://academic.oup.com/jid/article/218/5/673/4956225>

The text that needs to be addressed is in the Discussion section.

AU: The first sentence is from DHHS “the choice between an INSTI, PI, or NNRTI as the third drug in an initial ARV regimen should be guided by the regimen’s efficacy, barrier to resistance, adverse effects profile, convenience, comorbidities, concomitant medications, and the potential for drug-drug interactions” We have inserted the whole paragraph in brackets, and specified that the sentence is based on the DHHS guidelines

The second is “result in cross-resistance to dolutegravir and bictegravir. The reference you mention is now quoted (no. 36, Kuritzkes DR. Resistance to Dolutegravir—A Chink in the Armor? The Journal of Infectious Diseases, 2018, 218: 673–675)

In your revision ensure you cite all your sources (including your own works), and quote or rephrase any duplicated text outside the methods section. Further consideration is dependent on these concerns being addressed.

6.  Thank you for stating the following in the Competing Interests section:

MC has received honoraria as a speaker and/or advisor from  Abbott, Bayer, Cephalon, Gilead, Novartis and ViiV Healthcare, SGP  has received  research grants from Gilead Sciences, ViiV Healthcare, Abbott, MS&D, and  honoraria as a speaker from MS&D, Gilead Sciences, ViiV Healthcare, Abbvie, Janssen. These activities were not related to their work with this review

Please confirm that this does not alter your adherence to all PLOS ONE policies on sharing data and materials, by including the following statement: "This does not alter our adherence to  PLOS ONE policies on sharing data and materials.” (as detailed online in our guide for authors <http://journals.plos.org/plosone/s/competing-interests>).  If there are restrictions on sharing of data and/or materials, please state these. Please note that we cannot proceed with consideration of your article until this information has been declared.

Please include your updated Competing Interests statement in your cover letter; we will change the online submission form on your behalf.

AU: we have updated this as requested

Please know it is PLOS ONE policy for corresponding authors to declare, on behalf of all authors, all potential competing interests for the purposes of transparency. PLOS defines a competing interest as anything that interferes with, or could reasonably be perceived as interfering with, the full and objective presentation, peer review, editorial decision-making, or publication of research or non-research articles submitted to one of the journals. Competing interests can be financial or non-financial, professional, or personal. Competing interests can arise in relationship to an organization or another person. Please follow this link to our website for more details on competing interests: <http://journals.plos.org/plosone/s/competing-interests>

[Note: HTML markup is below. Please do not edit.]

5. Review Comments to the Author

Please use the space provided to explain your answers to the questions above. You may also include additional comments for the author, including concerns about dual publication, research ethics, or publication ethics. (Please upload your review as an attachment if it exceeds 20,000 characters)

Reviewer #1: There are several mislabeled items in the plots. Please mention if the authors performed tests for multiplicity.

AU: as we have specified below (comments), we have made consistent legends of figures and graph. We did not perform tests for multiplicity

Reviewer #2: The Authors performed a systematic review and meta-analysis of RCTs comparing dolutegravir-containing ART to non-dolutegravir containing ART in HIV-infected naive patients.The manuscript is well written and represents a very comprehensive analysis on this topic. The methodology is adequate and updated.
I have just one suggestion. Since the use of patients reported outcomes represent a more useful tool compared to clinician report of adverse events and they can explore outcomes such as patients' satisfaction with treatment, and considering the high virological response rates reported in some comparison between INSTI-based regimens in particular, it could be useful to include, when available, PROs as specific outcomes in the metanalysis (Whol, The Patient - Patient-Centered Outcomes Research, 2018).

AU: Thank you for this interesting observation, Unfortunately, as we have now specified in lines 369-371, measures of quality of life were rarely reported in the included studies, and we could not assess treatment satisfaction.

While revising your submission, please upload your figure files to the Preflight Analysis and Conversion Engine (PACE) digital diagnostic tool, <https://pacev2.apexcovantage.com/>. PACE helps ensure that figures meet PLOS requirements. To use PACE, you must first register as a user. Registration is free. Then, login and navigate to the UPLOAD tab, where you will find detailed instructions on how to use the tool. If you encounter any issues or have any questions when using PACE, please email us at [figures@plos.org](mailto:figures@plos.org). Please note that Supporting Information files do not need this step.

PONE-D-19-17880

Dolutegravir based antiretroviral therapy compared to other combined antiretroviral regimens for the treatment of HIV-infected naive patients: a systematic review and meta-analysis

Comments:

Define “naïve” in the Introduction

AU: Done (lineS 64-65)

There are only two authors. Was there an adjudicator in the data extraction process in case of disagreements?

AU: as specified on lines 110-111, agreement was achieved by discussion

90 ***Assessment of risk of bias and Heterogeneity***

Below line 90

Put here gradings of heterogeneity as in I^2^ = 0% indicating no heterogeneity

AU: Actually, the interpretation of I^2^ values is more complicated, and can be misleading; often, threshold of interpretation are: ≤25, low heterogeneity; >50, high heterogeneity (now specified in the text, line 94), but these values are completely arbitrary. So, as for other systematic reviews of our groups, we decided to use fixed effect model when I^2^ =0 and when values of I² are greater than zero, both fixed and random effects analyses were performed (see sentences on line 140-2 “We used the fixed-effect model when the I^2^ was=0,….”)

Restrict mentioning RevMan more than twice

AU: REV MAN is mentioned on line 132 and 145

Did the authors perform tests for heterogeneity?

AU: yes (see lines 118-121)

***Strategy for data synthesis***

104 We used aggregate data. The analysis has been conducted

Substitute the underlined with “was”

AU: done

107 random effects analyses are undertaken and any differences

Substitute the underlined with “performed”

AU: done

109 pre-specified subgroup analysis. We used risk difference as measure of effect. Review Manager 5.3 was used to analyze the data.

RevMan again?

AU: yes, for the second time

Table 2 . legend

Include RD

AU: done

203 adverse events and of adverse events requiring discontinuation of treatment was not significantly different in dolutegravir

What is the p-value for the “not significantly different “?

AU: the p values for any adverse events and adverse events requiring discontinuation have bee added to the text (line 273)

Kindly organize your Discussion so as to indicate summary of effects, clinical implications

Also mention of strengths and limitations of this study.

AU: summary effects and clinical implications are discussed (lines 321-348) and strengths and limits are now mentioned (lines 364-9)

156 Table 2 . Summary of the pooled outcome data. Results are provided for a 156 ll possible comparisons, and for

157 subgroups analyses

In column 5 of the P-values, the authors present several significant values

Did the authors correct for multiple comparisons?

AU: no, we did not.

The favours labels are opposite at the x-axis in Figures 3 and 5

Is this correct?

AU: yes, it is correct. In fig 4 thr RD is >0, which means that more people in DTG had VL<50 copies, a favourable outcome resulting in effect estimates to the right of the vertical line. In fig. 5 the RD is less than 0, which means that less people in DTG has treatment discontinuation, a favourable outcome resulting in effect estimates to the left of the vertical line

Suppl figures 1 and 2

Need to label DTG in legend

AU: we have made consistent legends of figures and graphs, using the entire words for dolutegravir and comparators

Supl Fig 4. Forest plot of comparison: DTG vs EFV, outcome: VL<50 48 wks

SINGLE 48 contributed 57.7% to the pooled RD, mention this in the text because it is an imbalance in weight contribution

AU: this is now mentioned in the text (lines 236-7)

Supl Fig 5. Forest plot of comparison: Dolutegravir vs comparators, outcome: VL<50 96 wks

The authors used random-effects here, why not fixed?

AU:because I^2^=31, and we chosed, starting from the protocol, to present the result of the fixed effect model when I^2^ =0

Supl Fig 6. Forest plot of comparison: DTG vs other according to baseline CD4 (cut off 200 CD4/ml) ,

outcome: % <50.

The authors used random-effects here even if the 9.3.1 plot is I^2^ = 0%. Please justify

AU: we used random effects model because in one of the subgroup there was evidence of heterogeneity; however, we performed analysis also using the fixed effect, and the results were much the same

Supl Fig 7. Forest plot of comparison: Dolutegravir vs comparators, outcome: Most common adverse

effects. Is the label favouring Dolutegravir same is DTG?

AU: as for fig 5, a RD <0 is a favourable outcome for AE. The 95 % CIs cross the vertical line for all the outcome analyzed
